# Methylomics analysis identifies ZNF671 as an epigenetically repressed novel tumor suppressor and a potential non-invasive biomarker for the detection of urothelial carcinoma

**DOI:** 10.18632/oncotarget.4986

**Published:** 2015-07-22

**Authors:** Chia-Ming Yeh, Pi-Che Chen, Hsiao-Yen Hsieh, Yeong-Chin Jou, Chang-Te Lin, Ming-Hsuan Tsai, Wen-Yu Huang, Yi-Ting Wang, Ru-Inn Lin, Szu-Shan Chen, Chun-Liang Tung, Shu-Fen Wu, De-Ching Chang, Cheng-Huang Shen, Cheng-Da Hsu, Michael W.Y. Chan

**Affiliations:** ^1^ Department of Life Science, National Chung Cheng University, Min-Hsiung, Chia-Yi, Taiwan; ^2^ Institute of Molecular Biology, National Chung Cheng University, Min-Hsiung, Chia-Yi, Taiwan; ^3^ Department of Urology, Ditmanson Medical Foundation Chia-Yi Christian Hospital, Chia-Yi, Taiwan; ^4^ Department of Pathology, Ditmanson Medical Foundation Chia-Yi Christian Hospital, Chia-Yi, Taiwan; ^5^ Department of Medical Research, Ditmanson Medical Foundation Chia-Yi Christian Hospital, Chia-Yi, Taiwan; ^6^ Departments of Radiation Oncology, Buddhist Dalin Tzu Chi General Hospital, Chia Yi, Taiwan

**Keywords:** urothelial carcinoma, DNA methylation, urine, ZNF671

## Abstract

The molecular mechanism underlying the lethal phenomenon of urothelial carcinoma (UC) tumor recurrence remains unresolved. Here, by methylation microarray, we identified promoter methylation of the zinc-finger protein gene, *ZNF671* in bladder UC tumor tissue samples, a finding that was independently validated by bisulphite pyrosequencing in cell lines and tissue samples. Subsequent assays including treatment with epigenetic depressive agents and *in vitro* methylation showed *ZNF671* methylation to result in its transcriptional repression. *ZNF671* re-expression in UC cell lines, via ectopic expression, inhibited tumor growth and invasion, in possible conjunction with downregulation of cancer stem cell markers (c-KIT, NANOG, OCT4). Clinically, high *ZNF671* methylation in UC tumor tissues (n=96; 63 bladder, 33 upper urinary tract) associated with tumor grade and poor locoregional disease-free survival. Quantitative MSP analysis in a training (n=97) and test (n=61) sets of voided urine samples from bladder UC patients revealed a sensitivity and specificity of 42%-48% and 89%-92.8%, respectively, for UC cancer detection. Moreover, combining DNA methylation of *ZNF671* and 2 other genes (*IRF8* and *sFRP1*) further increased the sensitivity to 96.2%, suggesting a possible three-gene UC biomarker. In summary, *ZNF671*, an epigenetically silenced novel tumor suppressor, represents a potential predictor for UC relapse and non-invasive biomarker that could assist in UC clinical decision-making.

## INTRODUCTION

Urothelial carcinoma (UC, also known as transitional cell carcinoma, TCC) is the second most common genitourinary malignancy and the ninth most common cancer in the world [1, 2]. UC is particularly common in Taiwan, where the highest incidence occurs in the southwestern coastal region [3]. The majority of UC tumors are found in the urinary bladder, and while upper urinary tract (ureter or renal pelvis) UC accounts for 5%-10% of all worldwide cases [4], its incidence in Taiwan is around 25% [5]. Although approximately 80% of UC tumors are non-muscle invasive, 70% of those will recur with muscle invasion [6, 7]. More notably, synchronous or metachronous UC through the urinary tract are not uncommon, thus necessitating long-term, repeated follow-up with highly invasive cystoscopy to monitor patients recurrence. To avoid this unpleasant procedure (and possibly, diminished patient compliance), alternative, non-invasive UC detection methods are urgently needed. Although conventional urine cytology remains the “gold standard” for non-invasive UC detection and disease monitoring, this method suffers from poor sensitivity ( < 34%) [8].

Accumulation of multiple genetic and epigenetic alternations that lead to the activation of proto-oncogenes and/or inactivation of tumor-suppressor genes (TSGs) is a ubiquitous event in human carcinogensis [9–11]. The intimate association of cancer and epigenetic aberrations, such as deviant DNA methylation and histone modifications, is reflective of the indispensability of epigenetics as a gene regulatory mechanism governing multi-/pluripotent cell fate commitment [12, 13]. As loss of differentiation is an essential and omnipresent event in carcinogenesis, reversal of dedifferentiating epigenetic aberrations could be highly effective for the therapy of several tumor types [14, 15].

In addition to the potential promise of epigenetic therapies, since altered chromatin (including aberrant methylomes) is one of most common molecular aberrations in human disease, methylated DNA represents an ideal bio-molecule for clinical diagnosis, due to its stability, presence in body fluids, and ease of detection [14, 16].

Previous reports, including our own, have identified numerous cancer-associated TSGs that are transcriptionally silenced by promoter DNA methylation [17–21]. However, the role of epigenetic alterations in the tumor progression and recurrence of urothelial carcinoma remains little explored. Consequently, we herein set forth to identify epigenetically silenced genes involved in UC tumor progression and recurrence, using methylomic screening of primary normal human urothelial cells (HUCs) and bladder UC tissue samples. That screening, and other assays, revealed one DNA methylation target, *ZNF671,* to be epigenetically silenced by promoter methylation in bladder UC. Our results demonstrate that *ZNF671* promoter methylation is frequently found in urothelial carcinoma and thus, represents a promising, non-invasive biomarker for the detection and guided therapy of urothelial carcinoma.

## RESULTS

### The promoter of the zinc-finger protein gene ZNF671 is frequently hypermethylated in bladder urothelial carcinoma

**Figure 1:**
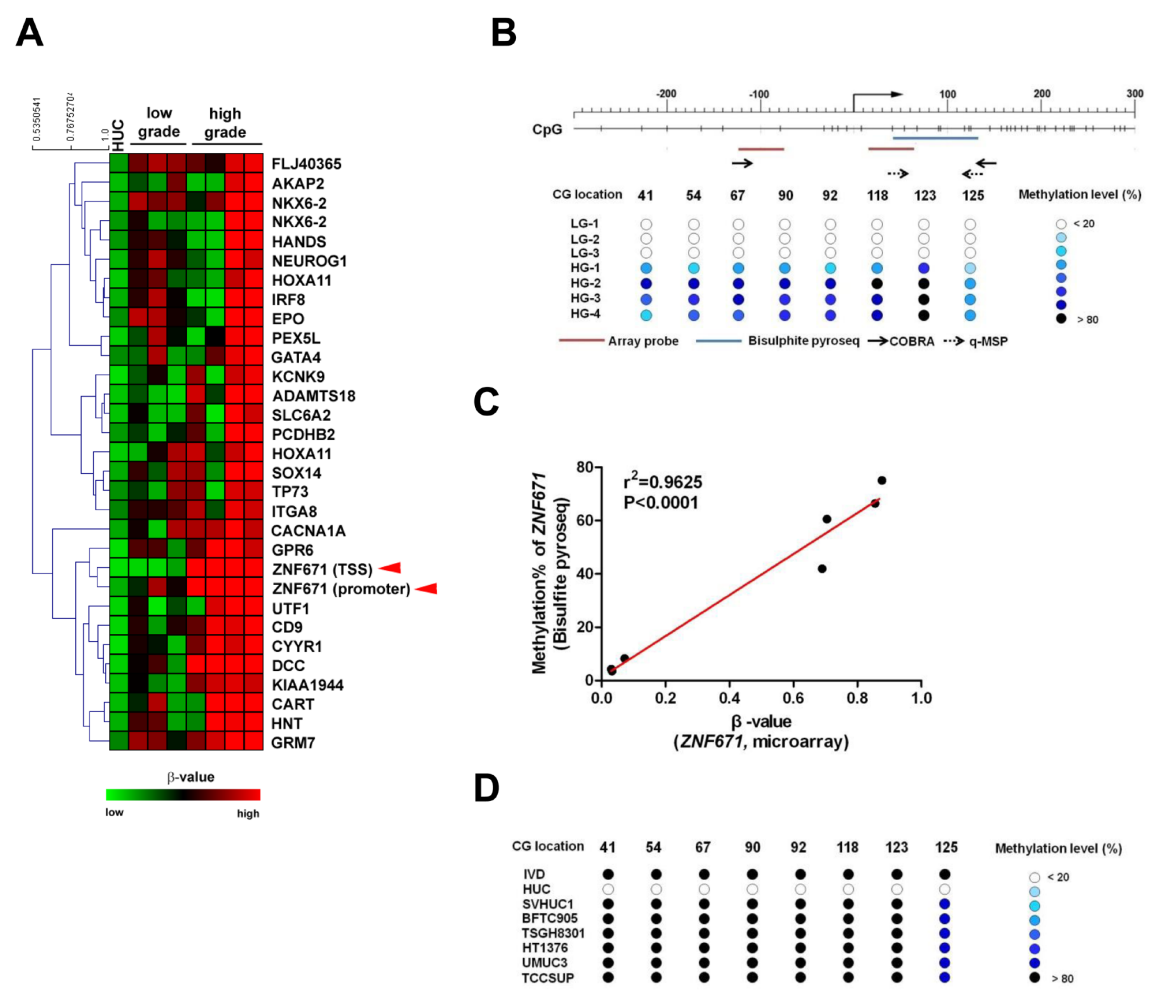
Methylation microarray identification of ZNF671 promoter DNA hypermethylation in bladder UC **A.** Heatmap showing DNA methylation levels (β-value) of selected probes in normal bladder urothelial (HUC, *n* = 1) and seven human bladder UC tissues (low-grade, *n* = 3; high-grade, *n* = 4) using an Illumina infinium 27K methylation array. One gene, *ZNF671* (red arrowheads) that showed promoter hypermethylation in bladder UC tissues was selected for further analysis. **B.** Upper panel shows the schematic diagram depicting the genomic structure and position of the CG sites (vertical dashes) in the *ZNF671* promoter region (from -271 to +290 with respect to the TSS). Right-angled arrow indicates the *ZNF671* gene transcriptional start site (TSS, defined as nucleotide 0). The location of the microarray probes (red horizontal line) at the TSS and promoter, bisulphite pyrosequencing (blue) and primers (small horizontal arrows) for COBRA (solid arrows) and qMSP (dashed arrows) are indicated. Lower panel shows the DNA methylation levels (as determined by bisulphite pyrosequencing) in the *ZNF671* promoter in DNA from 7 bladder UC tissues used in the microarray analysis. Numbers indicate the locations (nucleotide number with respect to the TSS) of specific CpGs examined. LG denotes low grade, while HG denotes high grade. **C.** Scatter plot showing the correlation between the β-value (from microarray) and methylation percentage (as determined by bisulphite pyrosequencing) of *ZNF671* CG sites in 7 bladder UC tissues. There was a significant correlation between β-value and methylation percentages (r^2^ = 0.9625, *P* < 0.0001). **D.** Methylation levels of bladder cancer cell lines as determined by bisulphite pyrosequencing. IVD (*in vitro* methylated DNA) was included as positive control.

We previously identified several tumor suppressors that are epigenetically silenced by promoter methylation in bladder UC. To identify additional tumor suppressor genes in bladder UC that might associate with tumor progression, and possibly be present in body fluids, we performed Illumina 27K CpG island methylation array on DNA from HUCs (primary normal human urothelial cells) and 7 bladder UC tissue samples (three low-grade and four high-grade). There were a total of 31 probes (representing 28 genes and three redundancies) that showed low β-values (β < 0.1, see Materials and Methods) in HUC cells but high β-values in tumor tissue samples (β-values: 0.2-0.5 in low grade samples; > 0.5 in high grade samples, Figure 1A, Supplementary Table 2). Subjection of this 28-gene set of differentially methylated genes to gene ontology analysis by DAVID [22] revealed over-representation of genes involved in transcriptional activity (Supplementary Table 3). One identified target, the *ZNF671* transcriptional repressor (Figure 1A, arrow head), caught our attention, as *ZNF671* was recently found epigenetically silenced by promoter DNA methylation in renal cell and cervical cancers [23, 24]. These findings are consistent with previous reports that other *ZNF* family proteins can act as tumor suppressors and are frequently found down-regulated by DNA methylation in multiple human cancer types [25–27]. However, the role of *ZNF671* in bladder UC has never been explored. Based on all of the above, *ZNF671* was selected for further analysis of its potential role in the formation and progression of urothelial carcinoma.

We first confirmed the microarray result by bisulphite pyrosequencing that showed a linear correlation between β-values (from the array) and DNA methylation levels of the promoter region of *ZNF671* in the same tumor tissue samples (Figure 1B, 1C). We also determined *ZNF671* methylation levels in several bladder UC cell lines by combined bisulphite restriction analysis (COBRA; Supplementary Figure 1A) [28] and bisulphite pyrosequencing (Figure 1D). Those assays showed that *ZNF671* promoter methylation was obvious in all bladder UC cell lines but not in normal bladder HUC cells (Supplementary Figure 1A). These results suggest that the *ZNF671* promoter is hypermethylated in bladder UC patient samples and cancer cell lines.

### ZNF671 expression is epigenetically repressed in bladder UC cells

**Figure 2:**
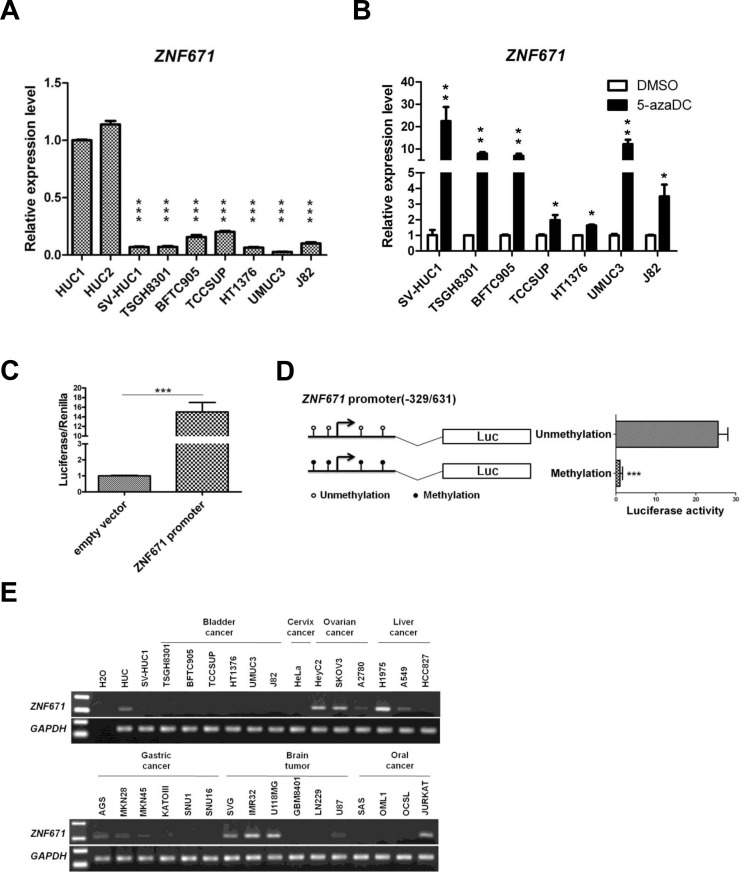
ZNF671 is epigenetically silenced in bladder UC cells **A.** *ZNF671* expression in two normal bladder urothelial (HUC) and bladder UC cell lines was determined by real time RT-PCR. All bladder UC cell lines, showed significant down-regulation of *ZNF671* expression, as compared to HUC cells. **B.** *ZNF671* expression in bladder UC cell lines after epigenetic treatment. Bladder UC cell lines treated with 5-aza-2′-deoxycytidine (5-azaDC) were examined for *ZNF671* re-expression by qRT-PCR. Epigenetic treatment significantly restored *ZNF671* expression. Each error bar represents standard deviations, as determined from triplicates assessments. **C.** Luciferase assay of the putative *ZNF671* promoter. A 960-bp region containing the putative *ZNF671* promoter (-329/631) was cloned into a pGL3 luciferase vector and transfected into 293T cells. As compared to the empty vector, the *ZNF671* promoter fusion construct exhibited luciferase activity. **D.** *In vitro* methylation assay of the *ZNF671* promoter. The *ZNF671* promoter region, with or without DNA methylation was cloned into pGL3 luciferase vectors and transfected into 293T cells. After 48 hours of transfection, cells were lysed and luciferase activity was analyzed. *In vitro* methylation of the *ZNF671* promoter significantly decreased luciferase activity. **E.** Endogenous *ZNF7671* expression was examined in different types of non-UC cancer cell lines by RT-PCR, showing that *ZNF671* was down-regulated in some, but not all, of the cancer cells examined.

Next, we investigated *ZNF671* expression in bladder UC cell lines using quantitative real-time RT-PCR amplification of RNA from bladder UC cell lines. Compared to HUC cells, *ZNF671* expression was significantly down-regulated in all bladder UC cell lines we examined (Figure 2A). To further access whether epigenetic repression, by DNA hypermethylation, was responsible for *ZNF671* transcriptional down-regulation, cells were treated with the demethylating agent, 5-aza-2′-deoxycytidine (5azaDC) (Figure 2B). Indeed, the addition of 5azaDC resulted in robust *ZNF671* re-expression of in all treated UC cells.

To examine, in more detail, how *ZNF671* expression is epigenetically controlled by promoter methylation, *in vitro* promoter methylation assays were performed. A 960-bp region (-329/631) containing the *ZNF671* promoter was cloned into a pGL3 luciferase reporter plasmid (Figure 2C), showing that *in vitro* methylation of the *ZNF671* promoter significantly (*P* < 0.05) decreased luciferase activity, as compared to the unmethylated control (Figure 2D).

As high *ZNF671* methylation levels were observed in bladder UC cell lines, and to exclude a possible “passenger event” due to selective pressure for UC cell survival in long-term culture [29], we examined *ZNF671* expression in several non-UC cancer cell lines including cervical, ovarian, gastric, liver, brain and oral cancer cell lines. In contrast to HUC cells, *ZNF671* was down-regulated in some, but not all of those non-UC cancer cell lines (Figure 2E). COBRA also confirmed that *ZNF671* promoter methylation correlated with gene down-regulation in these non-UC cells (Supplementary Figure 1B). Taken together, these results demonstrate that the zinc-finger protein gene, *ZNF671,* is epigenetically silenced in human urothelial carcinoma.

### ZNF671 ectopic expression in UC cells inhibits anchorage-dependent growth and invasion

**Figure 3:**
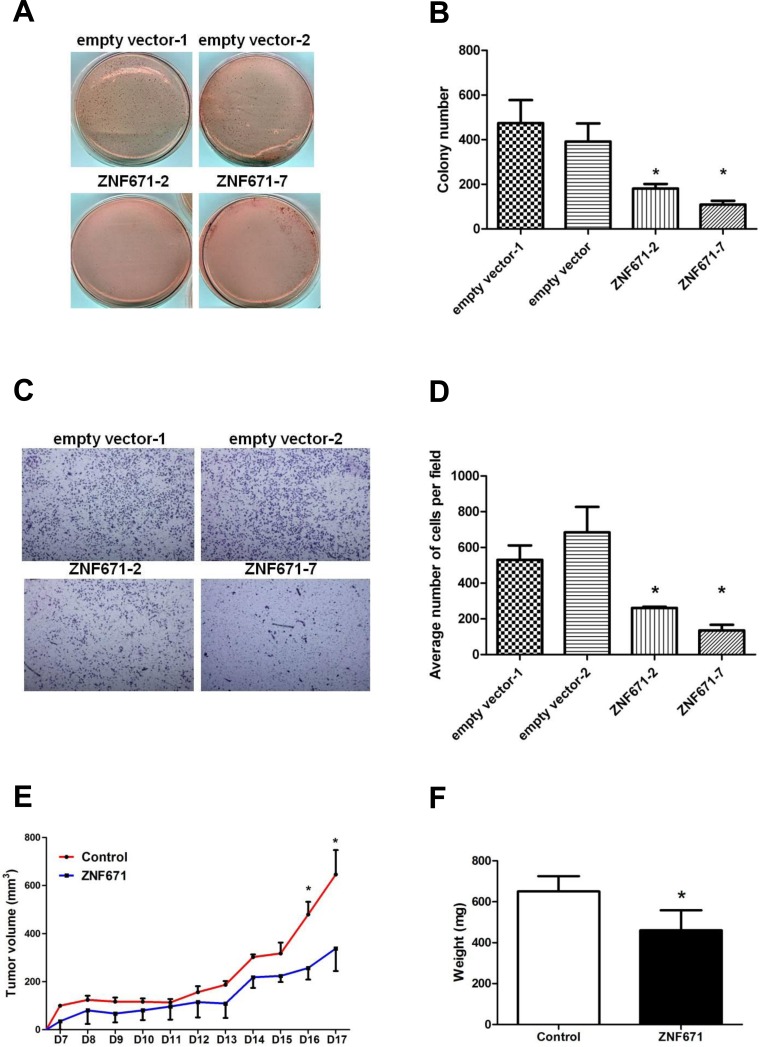
Ectopic expression of ZNF671 inhibits tumor growth and invasion in UMUC3 bladder UC cells **A.** Ectopic expression of *ZNF671* inhibited tumor growth as determined by soft agar assays. UMUC3 bladder UC cells transfected with empty or *ZNF671* expression vector (pcDNA3.1) were selected for soft agar growth assay. Cells stably overexpressing *ZNF671* (Supplementary Figure 2) generated significantly fewer colonies than the controls. **B.** Quantitative analysis of colony formation assay showing the number of colonies in the control and *ZNF671*-overexpression groups. **C.** UMUC3 bladder UC cells stably overexpressing *ZNF671* demonstrated significantly lower invasion ability than the control cells. **D.** Quantitative analysis of invasion assay. *ZNF671* overexpression inhibited tumor invasion in bladder UC cells. **E.** Restored *ZNF671* expression similarly inhibited tumor growth *in vivo.* Control or *ZNF671*-overexpressing cells were injected subcutaneously into nude mice. Tumor volumes, measured daily, showed significant differences between the control and *ZNF671*-overexpressing group upon and after day 16. **F.** Tumor weights were measured at the end of the experiments. Significant differences between tumor weights were noted when comparing the controls and *ZNF671*-overexpressing cells xenograft. *P < 0.05.

Having demonstrated that *ZNF671* is epigenetically silenced by DNA methylation, we then investigated a possible tumor suppressive role of *ZNF671* in bladder UC cells. Stable transfectants were selected from UMUC3 bladder UC cells transfected with a *ZNF671*-expression vector (*ZNF671* expression levels shown in Supplementary Figure 2). As compared to stable vector only-transfected cells, *ZNF671-*expressing UMUC3 bladder UC cells showed significant attenuation of anchorage-dependent growth (Figure 3A, 3B) and invasion (Figure 3C, 3D).

### Restoration of ZNF671 expression inhibits tumor growth *in vivo*

Based on our cell culture findings, we next examined the tumor suppressive effects of *ZNF671* in an *in vivo* xenograft mouse model. Nude mice subcutaneously injected with *ZNF671*-overexpressing UMUC3 cells grew tumors of significantly less tumor volume and weight, as compared to mice injected with UMUC3 cells stably transfected with the empty vector control (Figure 3E, 3F). Taken together, these *in vitro* and *in vivo* results strongly suggest *ZNF671* to be a potential tumor suppressor in advanced human bladder UC.

### Over-expression of ZNF671 down-regulates the expression of cancer stem cell markers in bladder urothelial carcinoma

**Figure 4:**
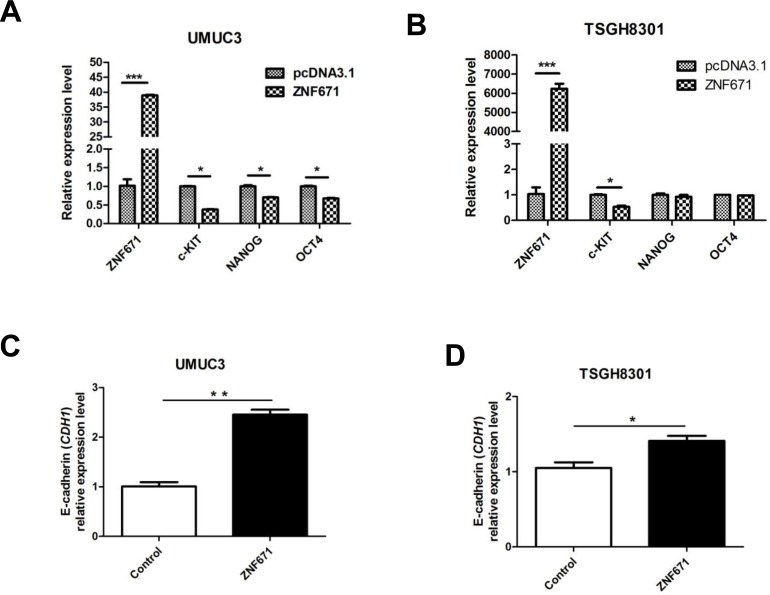
ZNF671 expression alters the expression of cancer stem cell markers Expression of stem cell markers (*c-KIT*, *NANOG* and *OCT4*) were detected by qRT-PCR in **A.** UMUC3; **B.** TSGH8301 bladder UC cells. Relative expression of E-cadherin (*CDH1*) in *ZNF671-*overexpressing **C.** UMUC3 and **D.** TSGH8301 bladder UC cell lines. *ZNF671* overexpression resulted in up-regulation of E-cadherin (*CDH1*) in *ZNF671*-overexpressing cells, as compared to (vector only-transfected) controls. *P < 0.05, **P < 0.01, ***P < 0.001.

A current consensus asserts that the tumor invasion-associated epithelial-mesenchymal transition (EMT) intimately correlates with cancer stem cell (CSC) phenotypes [30]. Such “phenotypic plasticity” that allows for transitions between different levels of cellular differentiation is governed by genome-wide epigenetic modifications [31, 32]. To assess the possibility that *ZNF671* tumor suppressive activity might relate to disruption of cancer “stemness” or EMT phenotypes, we examined the effects of ZNF671 on the expression of CSC markers. Interestingly, *ZNF671* overexpression significantly decreased the expression of three stem cell markers, *c-KIT*, *NANOG*, and *OCT4* [33] in UMUC3 (Figure 4A) but only one such marker (*c-KIT*) in TSGH8301 bladder UC cells (Figure 4B). Importantly, significant up-regulation of *CDH1* (E-cadherin) was observed in both *ZNF671*-overexpressing UMUC3 and TSGH8301 cells (Figure 4C, 4D). Taken together, these results support the postulate that *ZNF671* could inhibit tumor growth and invasion through down-regulation of determinants of dedifferentiation.

### ZNF671 is epigenetically silenced in UC patient samples

**Figure 5:**
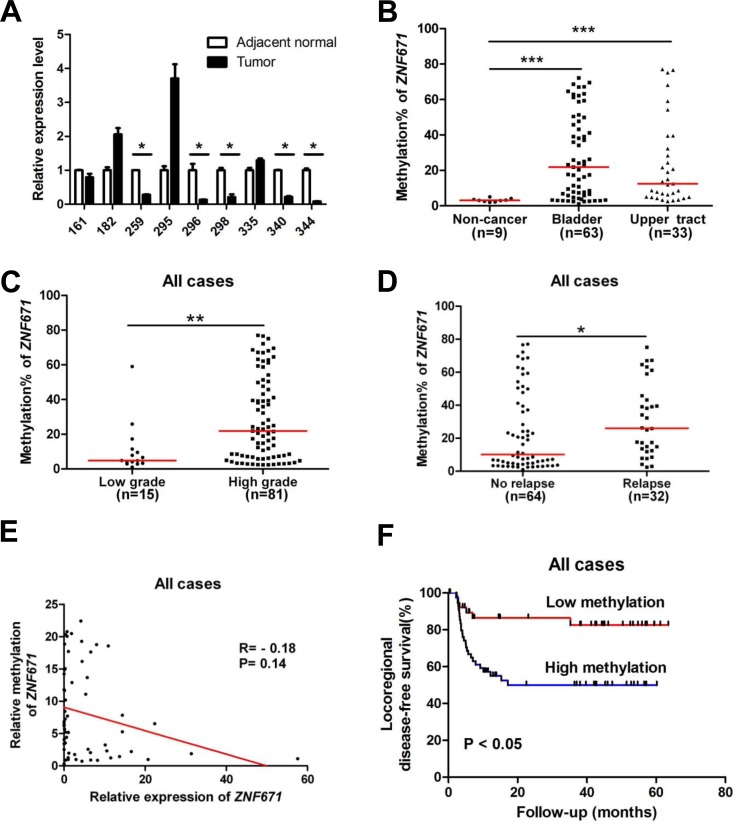
ZNF671 is epigenetically silenced by promoter hypermethylation in UC patients, and associates with tumor recurrence **A.** *ZNF671* relative expression in bladder UC tumor tissues, and their corresponding adjacent normal from the same patient, as determined by qRT-PCR. Down-regulation of *ZNF671* was observed in five UC tumor patients, as compared to their adjacent normal tissues. *P < 0.05. *ZNF671* methylation levels of in 96 UC patient tissue samples were determined by bisulfite pyrosequencing. Higher *ZNF671* methylation levels were observed in **B.** cancer tissues from bladder or upper urinary tract neoplasms, as compared to 9 non-cancerous tissues, **C.** patient tissues with higher tumor grades and **D.** UC patients with relapse, *P < 0.05, **P < 0.01. **E.** Scatter plot showing the relationship between relative *ZNF671* methylation and expression in 60 UC patient tissue samples (bladder, *n* = 38, upper urinary tract, *n* = 22), relative to *ZNF671* methylation and expression of in HUC (which was set to 1). As shown, a trend showed an inverse relationship between *ZNF671* methylation and expression in this group of patient tumor tissue samples. **F.** Kaplan-Meier analysis of tumor tissues *ZNF671* methylation for locoregional disease-free survival of UC patients. Patients with higher *ZNF671* methylation ( > 11% methylation, please refer to Material and Methods) demonstrated shorter DFS than patients with lower methylation. Log-rank test, **P < 0.01.

The above experiments demonstrate that *ZNF671* is likely a tumor suppressor that is transcriptionally suppressed by promoter DNA methylation in bladder UC cell lines. To examine the possible clinical significance of *ZNF671* methylation to UC progression, we investigated the promoter methylation and expression of *ZNF671* in UC patient samples. First, we compared the relative expression of *ZNF671* in nine bladder UC tissues, and their corresponding adjacent normal tissues. *ZNF671* expression was significantly down-regulated in five tumor tissue samples as compared to their adjacent normal counterparts (Figure 5A). We further examined the methylation status of *ZNF671* by bisulphite pyrosequencing in 63 bladder UC tissues and 9 adjacent normal tissues (Table 1). To assess possible detection of *ZNF671* methylation in various UC subsites, 33 upper urinary tract UC tissue samples originating from the ureter or renal pelvis, were also procured for analysis (Table 1). *ZNF671* methylation in cancer tissues was significantly higher than in adjacent non-cancerous tissues (Figure 5B, *P* = 0.0001). Importantly, there was no significant difference between *ZNF671* methylation in UC tissue samples from the bladder *vs*. upper urinary tract (Figure 5B). Moreover, further analysis showed that *ZNF671* methylation was significantly elevated in high-grade, but not low-grade, cancer tissues (Figure 5C, Table 2, *P* = 0.003). Such tumor grade trends could also be observed in bladder or upper urinary tract UC tumor tissues samples (Supplementary Figure 3A, 3B). Importantly, although not significant, there was an inverse relationship between *ZNF7671* promoter methylation and expression in UC patient tumor samples (*n* = 60, Figure 5E, Spearman R = −0.18, *P* = 0.14). Interestingly, we also observed a group of patients (*n* = 7) showing the opposite correlation, i.e., low *ZNF671* expression and concomitant with low DNA methylation, thus suggesting that other genetic or epigenetic mechanisms contribute to *ZNF671* down-regulation in this specific sub-group. By removing this sub-group from the analysis, *ZNF671* promoter methylation showed a statistically significant inverse relationship with expression (Supplementary Figure 4, R = −0.32, *P* = 0.01). Taken together, these results suggest that *ZNF671* is epigenetically silenced by promoter methylation, with progressively increased methylation positively correlating with increased UC patient tumor grade.

**Table 1:** Summary of clinical-pathological data of UC tissue and urine samples

	Tissue samples		Urine samples/Training set		Urine samples/Test set	
	Cancer (n = 96)	Adjacent normal (n = 9)	Cancer (n = 69)	Non-cancer (n = 28)	Cancer (n = 33)	Non-cancer (n = 28)
Age	69.52 ± 11.94^4^	78.71 ± 6.57	68.55 ± 10.15	56.03 ± 16.32	70.36 ± 9.74	60.85 ± 14.7
Gender						
Male	68^5^	6	47	18	17	20
Female	28	3	22	10	16	8
Location						
Bladder	63					
Upper tract^1^	33					
Histological Grade^2^						
Low Grade	15		20		6	
High Grade	81		49		27	
Pathological Stage						
pT1	54		54		21	
pT2–4	42		15		12	
Primary/Recurrent						
Primary	83		43		12	
Recurrent	13		25		20	
Unknown	0		1		1	
Relapse						
Yes	32					
No	64					
Treatment						
TURBT	60					
Non-TURBT^3^	36					
Methylation (%)	26.07	3.19	22.07	1.31	29.45	3.86

1 Upper urinary tract UC originating from the ureter or renal pelvis;

2 Grading, low grade: G1; high grade: G2–3;

3 Radial cystectomy (*n* = 3) or nephroureterectomy with bladder cuff excision (*n* = 33);

4 Mean ± SD

5 Number of cases except otherwise stated

### ZNF671 methylation associates with shorter locoregional disease-free survival

Based on our observed relationship between increased *ZNF671* hyper-methylation and tumor grade, we examined possible association of *ZNF671* methylation levels with UC tumor recurrence, showing that *ZNF671* methylation was significantly higher in relapsed tumor tissue samples (Figure 5D, Table 2). Further sub-group analysis yielded similar results in bladder, but not in upper urinary tract UC tumor tissue samples (Supplementary Figure 3C, 3D, Supplementary Table 4). Although recurrent tumor tissue samples tended to be of higher tumor grade (Supplementary Figure 5A, 5B), there was no difference in *ZNF671* methylation between primary and recurrent samples (Supplementary Figure 5C, 5D), likely due to the relatively small sample size of recurrent cases.

**Table 2:** Correlation between *ZNF671* methylation and clinical-pathological data in 96 UC samples

	Methylation (%)	P
**Age**		
< 60 years	24.15 ± 26.35^2^ (19/96)	0.42
≥ 60 years	26.55 ± 22.92 (77/96)	
**Gender**		
Male	26.80 ± 24.00 (68/96)	0.62
Female	24.32 ± 22.61 (28/96)	
**Histological Grade^1^**		
Low grade	11.05 ± 14.79 (15/96)	**0.003**
High grade	28.86 ± 23.83 (81/96)	
**Pathological Stage**		
pT1	29.64 ± 25.70 (54/96)	0.38
pT2–4	21.49 ± 19.72 (42/96)	
**Primary/Recurrent**		
Primary	24.89 ± 23.05 (83/96)	0.24
Recurrent	33.61 ± 26.00 (13/96)	
**Relapse**		
Yes	31.25 ± 22.09 (32/96)	**0.03**
No	23.49 ± 23.94 (64/96)	
**Methylation**		
Low	5.12 ± 2.40 (39/96)	**< 0.001**
High	40.41 ± 20.47 (57/96)	

1 Grading, low grade: G1; high grade: G2–3;

2 Mean ± SD.

The correlation between UC tumor tissue *ZNF671* hypermethylation and locoregional disease-free survival was also analyzed. Kaplan-Meier analysis demonstrated that patients with higher *ZNF671* methylation (a cutoff value of 11%, see Material and Methods) demonstrated shorter locoregional disease-free survival than in patients with lower methylation (Figure 5F, *P* < 0.05). Multivariate analysis also confirmed that *ZNF671* methylation was an independent risk factor for predicting recurrence (HR, 3.742; 95% CI, 1.464 – 9.562; *P* < 0.01; Table 3). Importantly, subgroup analysis also showed that the above-mentioned trend could also be observed in bladder or upper urinary tract UC tissues samples (Supplementary Figure 3E, 3F; Supplementary Table 5, 6).

**Table 3:** Hazard ratios for recurrence according to predictive factors, in 96 UC tissue samples

	*HR(95% CI); P value*		
	Univariate analysis	Multivariate analysis
**Age**		
< 60 vs ≥ 60	1.608(0.619–4.178);0.329	NA
**Gender**		
Male vs Female	0.405(0.156–1.053);0.064	NA
**Histological Grade**		
Low vs High grade	1.165(0.449–3.027);0.753	0.787(0.272–2.279);0.659
**Pathological Stage**		
pT1 vs pT2–4	0.764(0.374–1.564);0.462	0.873(0.380–2.003);0.749
**Primary/Recurrent**		
Primary vs Recurrent	1.693(0.651–4.403);0.280	1.627(0.599–4.418);0.339
**Treatment**		
TURBT vs Non-TURBT	0.670(0.317–1.414);0.293	0.892(0.374–2.129);0.797
***ZNF671* Methylation**		
Low vs High	3.570(1.467–8.687); **< 0.01**	3.742(1.464–9.562); **< 0.01**

Abbreviation: HR, hazard ratio; 95% CI, 95% confidence interval;

TURBT, transurethral resection of bladder tumor

### ZNF671 methylation as a non-invasive UC biomarker in voided urine

**Figure 6:**
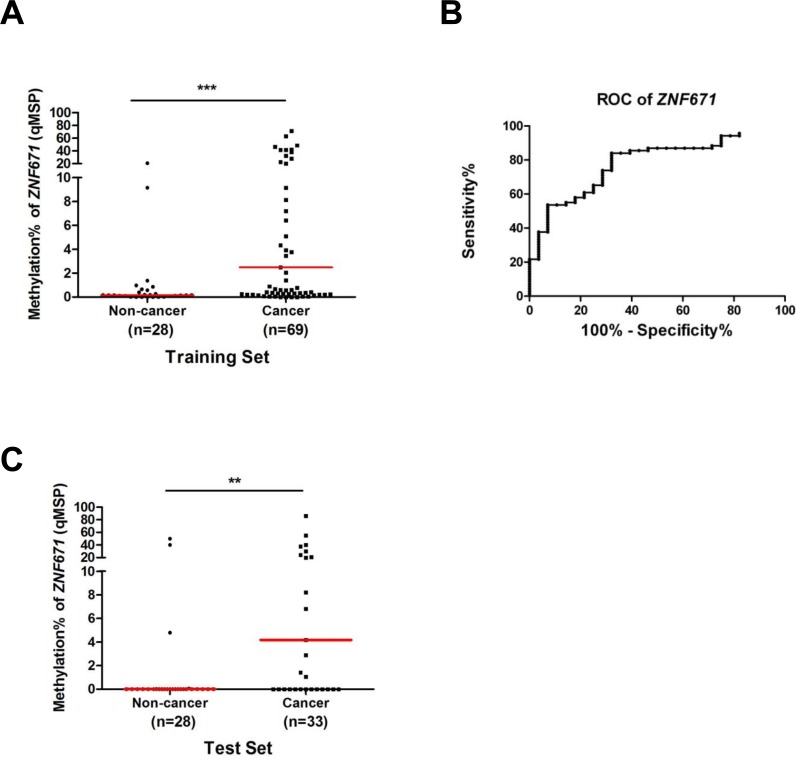
Quantitative MSP (qMSP) analysis of ZNF671 methylation in voided urine samples from bladder UC patients **A.** As a training set, methylation of *ZNF671* was determined by quantitative methylation specific PCR (qMSP) in voided urine samples from non-cancerous control (*n* = 28) and bladder UC patients (*n* = 69). Compared to the non-cancerous controls, higher *ZNF671* methylation levels were detected in cancer samples. ***P < 0.001. **B.** A receiver operating characteristic curve (ROC) of *ZNF671* methylation in urine samples from 28 non-cancer controls and 69 bladder UC patients. According to the area under the curve (AUC = 0.783)*,* methylation of *ZNF671* showed excellent discrimination for bladder UC. **C.** The predictive power of *ZNF671* methylation was determined in another set of 63 urine samples (from 28 healthy control individuals and 33 UC patients) for a single-blind qMSP analysis. Similar to above, compared to the non-cancerous controls, higher *ZNF671* methylation levels were also detected in cancer samples. **P < 0.01. The sensitivity and specificity of *ZNF671* methylation can be found in Table 4.

Having demonstrated that the potential tumor suppressor *ZNF671* is epigenetically silenced in UC, and in consideration of other cancer biomarkers found in human urine [17, 34], we assessed the feasibility of using *ZNF671* methylation as a biomarker for non-invasive cancer detection in urine. We first collected voided urine from 69 recruited bladder UC patients and 28 healthy control individuals as a training set (Table 1). DNA extracted from urine sediments were subjected to quantitative real-time methylation specific PCR (qMSP) analysis. As compared to the control samples, higher *ZNF671* methylation levels were detected in UC patients’ urine samples (Figure 6A, *P* < 0.001). Based on the cutoff value generated by the area under the receiver operating characteristic curve (ROC, AUC = 0.783, a cutoff value of 5.37%, Figure 6B), we observed methylated *ZNF671* to exhibit a sensitivity of 42% and a specificity of 92.8% for UC detection in urine (Table 4).

**Table 4:** Sensitivity and specificity of *ZNF671* methylation in cancer detection using voided urine samples

	Training set	Test set
**Sample size**		
Cancer	69	33
Non-cancer	28	28
**Sensitivity(%)**		
All cases	42% (29/69)	48% (16/33)
Low Grade	45% (9/20)	33% (2/6)
High Grade	40% (20/49)	52% (14/27)
Primary	41.8%(18/43)	58.3%(7/12)
Recurrent	44.0%(11/25)	45.0%(9/20)
**Specificity(%)**	92.8%	89%
**Positive predictive value (%)**	93.5%	84.2%
**Negative predictive value (%)**	39.3%	59.5%

To further examine the predictive power of *ZNF671* methylation for non-invasive cancer detection, we obtained another 61 urine samples (33 bladder UC and 28 non-cancer) as a test set for single-blind analysis (Table 1). Similarly, higher *ZNF671* methylation levels were detected in UC urine samples, as compared to urine from the control subjects (Figure 6C, *P* < 0.01). Using a similar cutoff as the training set, a sensitivity of 48% and a specificity of 89% for cancer detection were obtained (Table 4). These results suggest that *ZNF671* methylation may serve as a non-invasive biomarker for bladder UC detection using urine.

### A multi-methylation marker panel further increases the sensitivity of bladder UC detection

Our previous study demonstrated that a combined panel of methylated markers could substantially increase the sensitivity of the bladder UC detection in urine [18]. We therefore examined the sensitivity and specificity of cancer detection in urine by combining methylated *ZNF671* with methylation of two other TSGs (*IRF8* and *sFRP1*) using a previously published urine sample cohort (26 UCs and 19 non-cancerous controls) [18]. While the sensitivity and specificity of cancer detection using *ZNF671* methylation alone was 57.7% and 89.5% (Table 5), combining hypermethylation of *ZNF671* with that of *IRF8* and *SFRP1* increased the sensitivity to 96.2% (Table 5). Notably, the sensitivity of this marker panel for detection of high-grade samples could be up to 100% (Table 5). Taken together, these results imply that combining *ZNF671* DNA methylation with other methylated genes could represent a sensitive biomarker panel for non-invasive bladder UC detection using urine.

**Table 5:** Sensitivity and specificity of cancer detection using voided urine samples

	*IRF8*	*SFRP1*	*ZNF671*	*IRF8* or *SFRP1*	*ZNF671* or *IRF8*	*ZNF671* or *SFRP1*	^a^Marker panel
**Sensitivity(%)**							
All cases (*n* = 26)	61.5%	50.0%	57.7%	88.4%	80.8%	84.6%	96.2%
Low grade (*n* = 10)	50.0%	60.0%	40.0%	90.0%	60.0%	80.0%	90.0%
High grade (*n* = 16)	68.8%	43.8%	68.8%	87.5%	94.1%	87.5%	100%
Primary(*n* = 22)	68.2%	45.4%	54.5%	86.3%	81.8%	81.8%	95.4%
Recurrent(*n* = 4)	25.0%	75.0%	75.0%	100%	75.0%	100%	100%
**Specificity(%)**	94.7%	94.7%	89.5%	89.5%	84.2%	89.5%	84.2%
**Positive predictive value (%)**	94.1%	92.8%	88.2%	92.0%	87.5%	91.6%	92.6%
**Negative predictive value(%)**	64.2%	58.0%	60.0%	85.0%	76.2%	80.9%	94.4%

a Any one of these genes (*IRF8, SFRP1* or *ZNF671*) showed methylation in urine samples.

## DISCUSSION

Although urothelial carcinoma (UC) patients have a low mortality rate, the high recurrence rate of this cancer type requires long-term follow-up with repeated cystoscopy for monitoring return disease. Thus, a better understanding of the molecular carcinogenesis of UC tumor progression may help in developing novel, less invasive diagnostic markers. In this study, by using various DNA methylation assessments, we identified a novel tumor suppressor gene, *ZNF671* that is epigenetically silenced by promoter DNA methylation in UC.

ZNF671, which contains C2H2-type zinc fingers (ZFs) and a Krüppel associated box (KRAB) domain, is a member of the KRAB-ZF (KRAB-ZFP) family of mammalian transcriptional repressors [35–37]. Through recruitment of KRAB-associated protein-1 (KAP1) and other co-repressors, KRAB-ZFPs form heterochromatin with HP1, SETDB1, and various histone deacetylases (HDACs) to epigenetically silence their targets [37–41]. Furthermore, KRAB-ZFPs are also the largest group of transcription factors regulating cell differentiation, proliferation, apoptosis, tumor suppression, and neoplastic transformation [25, 42–44]. Previous studies demonstrated that some of these ZNF proteins are tumor suppressors that are epigenetically silenced by DNA methylation in multiple human cancers [25–27]. For example, *ZNF23* can enhance the expression of p27^KIP1^ to inhibit cancer cell growth [45], while *ZNF668* can enhance the stability of the p53 tumor suppressor by preventing MDM2-mediated p53 ubiquitination and its subsequent proteosomal degradation, in breast cancer [46]. While *ZNF382* and *ZNF545* are frequently down-regulated by promoter methylation, their expression can inhibit colony formation, proliferation and induce apoptosis via repression of the NF-kB and AP-1 signaling pathways in multiple tumors [25, 26].

Although *ZNF671* was previously found to be silenced by promoter methylation in renal cell and cervical carcinomas [23, 24], its role in UC has never been explored. In this study, we found that *ZNF671* was epigenetically silenced by promoter hypermethylation in UC cell lines and patient tumor tissue samples. Moreover, *ZNF671* methylation was significantly higher in tumor tissues, as compared to their adjacent normal tissues, and also significantly correlated with higher tumor grade and tumor relapse, in addition to associating with shortened locoregional disease-free survival (Figure 5). It is also noteworthy to point out that we observed a sub-group of UC patients with low *ZNF671* expression and low *ZNF671* methylation, suggesting that the role of other epigenetic modifications and/or genetic alterations contribute to *ZNF671* gene regulation.

We also observed *ZNF671* epigenetic repression in patient tumors of the upper urinary tract, which in Taiwan is a more common site for this malignancy [5], in addition to significant association with high tumor grade and shorter locoregional disease-free survival. Currently, the sensitivity of diagnostic tool for this UC is known to be low [47, 48]. Thus, a role for *ZNF671* methylation as a non-invasive biomarker for this disease deserves further investigation.

Our functional studies additionally showed that *ZNF671* overexpression inhibits UC invasion and growth both *in vitro*, and *in vivo* (in xenograft mouse models). Furthermore, recent studies showed that the transcriptionally repressive ZNF KRAB domain can recruit KAP1, another transcriptional co-repressor that cooperates in epigenetically silencing KRAB-ZNF targets [49, 50]. More recently, Lin *et al*. demonstrated that ZBRK1, a KRAB-ZFP could suppress tumor invasion by recruiting KAP1 in cervical cancer, as loss of ZBRK1 resulted in KAP1 up-regulation and tumor migration and invasion [51]. Further study of the role of KAP1 in the tumor suppressive function of ZNF671, however, is needed.

Previous reports, including ours, demonstrate that detection of DNA methylation in exfoliated cells from the urine of bladder UC patients represents a useful and sensitive biomarker for cancer detection [14, 17, 52, 53]. Remarkably, a multiple methylation marker panel can greatly enhance the sensitivity of cancer detection [18, 34], and the sensitivity of methylation markers is much higher than that of urine cytology in cancer detection especially for low-grade tumors [17, 34]. For example, in one large cohort study, urine cytology exhibited an overall sensitivity of 38% for bladder UC detection, while the sensitivity for low-grade tumors was only 11.9% [54]. In the current study, using qMSP assay, we found that *ZNF671* methylation exhibited a sensitivity of around 45% (42%-48%) in detecting bladder UC in urine, and that combining methylated *ZNF671*, *IRF8* and *sFRP1* increased the overall sensitivity for cancer detection in urine, to 96.2% (overall disease), 90% (low-grade disease) and remarkably 100% for high-grade tumors (Table 5). These results suggest that combining *ZNF671* with other methylation markers can result in highly sensitive methylation marker panels for possible non-invasive diagnosis of bladder urothelial carcinoma in voided urine. However, further experiments with larger sample sizes are needed to examine the predictive power of such methylation marker panels.

We must acknowledge several limitations of the current study. First, as a retrospective study, selection bias may exist and cannot be totally avoided even after multivariate analysis adjustment. Second, this sample cohort failed to demonstrate any association between tumor recurrence and tumor grade or stage, each of which are established prognostic factors for UC recurrence. This shortcoming may be attributed to the relatively small samples size of low-grade tumor samples. Therefore, prospective studies, with a more balanced sample-distribution should be conducted to confirm any hypothesized associations. Third, we have not analyzed *ZNF671* methylation in any voided urine samples from upper urinary tract UC patient specimens. Thus, the possible role of *ZNF671* methylation as a non-invasive biomarker for all UC sub-types requires further investigation.

In conclusion, *ZNF671* is a potential tumor suppressor that is epigenetically silenced by promoter methylation in bladder urothelial carcinoma. Methylation of *ZNF671* represents a novel epigenetic biomarker candidate for the non-invasive diagnosis of bladder UC. Moreover, a combination of *ZNF671*, *IRF8*, and *SFRP1* represents a sensitive candidate methylation marker panel for the detection of bladder UC in urine. Such biomarkers could serve as surrogates for excluding specific patient subgroups from regular cystoscopy, and could also help guide clinical decision-making by indicating tumor grade, recurrence or therapeutic response.

## MATERIALS AND METHODS

### Cell culture and epigenetic treatment

Human urothelial cell (HUC) were maintained in urothelial cell medium (UCM, Sciencell Research Laboratories, Carlsbad, CA). SV-HUC-1 cells were derived by transducing simian virus 40 (SV40) into normal human uroepithelial cells (HUC), as described previously [55], and maintained in F12 Nutrient Mixture (GIBCO, Grand Island, NY), supplemented with 10% fetal bovine serum (FBS) (Invitrogen, Carlsbad, CA) and 50 units/ml of penicillin/streptomycin (Invitrogen). TSGH8301 and BFTC905 cells were maintained in RPMI 1640 (GIBCO) supplemented with 10% fetal bovine serum (FBS) (Invitrogen), while TCCSUP cells were cultured in DMEM (GIBCO) supplemented with 10% FBS. UMUC3 and HT1376 cells were grown in MEM (GIBCO) supplemented with 10% FBS, 1% NEAA, 1uM sodium pyruvate. The transformed human embryonic kidney cell line (293T) was maintained in DMEM (GIBCO) supplemented with 10% FBS. All cells were incubated in 37°C at 5% CO_2_. For demethylation treatment, cells were seeded in a 60-mm plates and treated with 0.5 μM 5′-aza-2′-deoxycytidine (5azaDC, Sigma, St. Louis, MO) for 3 days. Culture media and drugs were replenished every 24 hr. Cells were then lysed and harvested for RNA analysis by reverse transcription and PCR.

### Patient samples

All patient samples were collected from the Ditmanson Medical Foundation Chia-Yi Christian Hospital, Chia-Yi, Taiwan. Seven bladder UC tissue samples (3 low-grade and 4 high-grade) from bladder UC patients were collected for Illumina 27K CpG island methylation array analysis. Another 63 bladder UC tissue samples and 9 cancer-adjacent normal tissues were collected for analysis. To examine whether *ZNF671* methylation could also be detected in UC in the upper urinary tract, 33 UC tissue samples from the ureter or renal pelvis were also procured for analysis. The clinical-pathological data of all samples is summarized in Table 1. All tissue samples were acquired from either transurethral resection (TURBT) or radical surgery. Patients were then followed up by either cystoscopy or radiographic detection (CT or MRI) for recurrence. For biomarker assessments, voided urine samples from 69 bladder UC patients and 28 non-cancer individuals were collected as a training set. Another voided urine samples from 33 bladder UC patients and 28 non-cancer individuals comprised a blinded test set. All human subjects assessments were approved by the Institutional Review Board (IRB) of the Ditmanson Medical Foundation Chia-Yi Christian Hospital, Taiwan.

### DNA extraction

DNA was extracted from cells and frozen tissues using Genomic DNA Mini Kit (Geneaid, Taiwan), according to the manufacturer's protocol. For urine samples, 50 ml of voided urine was centrifuged at 3,000 rpm for 10 min to obtain the sediment. The pellets were then washed 3 times with cold 1X PBS followed by centrifugation at 3,000 rpm for 10 min. DNA was then isolated using Genomic DNA Mini Kits (Geneaid), according to the manufacturer's protocol.

### Extraction of RNA and quantitative RT-PCR

RNA extraction was performed using TRizol reagent (Invitrogen) according to the manufacturer's protocol. To remove potential contaminating DNA from the complementary DNA, 1 μg of total RNA was treated with DNase I (Amplification Grade, Invitrogen) prior to reverse transcription. First-strand cDNA synthesis was carried out using MMLV High Performance Reverse Transcriptase (Epicentre, Chicago, IL). The real-time PCR reactions were performed on an ABI Step-One real-time PCR system (Applied Biosystems, Foster City, CA) with specific primers (Supplementary Table 1). Relative gene expression was determined by comparing the threshold cycle of the test gene against the Ct value of *GAPDH* in a given sample (i.e., the comparative Ct method).

### Bisulphite conversion and combined bisulphite restriction analysis (COBRA)

DNA was bisulphite modified using EZ DNA methylation kits (ZYMO Research, Orange, CA) according to the manufacturer's protocol as previously described [56]. For COBRA analysis [28], 4 μl of bisulphite converted DNA was first amplified using specific primers (Supplementary Table 1) targeting various *ZNF671* promoter regions, followed by digestion with 20 U of AciI (GGCG) (New England Biolabs, Ipswich, MA). *In-vitro* methylated DNA (IVD, Merck Millipore, Billerica, MA) was used as a positive control for methylation, and water was used as a negative control. The digested products were then separated on 1.5% agarose gels for visualization.

### Infinium microarray DNA methylation analysis

Bisulphite-modified DNA was subjected to methylation analysis using an Illumina Infinium Human Methylation27 microarray (Illumina, San Diego, CA), as previously described [57]. The methylation level of each probe (β-value) was defined by the intensity of the methylated allele (M) / (intensity of the unmethylated allele (U) + intensity of the methylated allele (M) + 100). Selection of differentially methylated probes used the following inclusion criteria: 1) probes were present in “CpG islands”, regions with much higher than normal CG content; 2) probes having mean β-values of < 0.5 in HUC cells and > 0.5 for tumor samples; and 3) a minimum difference of mean β-values ≥0.2 between tumor samples and HUC were obtained.

### Real-time quantitative methylation-specific PCR (qMSP) of urine sample DNA

For detection of *ZNF671* methylation in urine samples, DNA was extracted (see above) and quantitative real-time methylation-specific PCR (qMSP) was performed as previously described [18]. In brief, 4 μl of bisulphite converted DNA was subjected to real time MSP within specific promoter *ZNF671* regions using distinct primer sets (Supplementary Table 1) in an ABI Step One real time PCR system (ABI). *In-vitro* methylated DNA (IVD) (Merck Millipore, Billerica, MA) was used as a positive control for methylation. *β-actin* (*ACTB*) was used to normalize the experimental samples to the input DNA. Methylation levels were determined by the threshold cycle number (Ct) for each sample against a standard curve generated by an IVD-MSP cloned fragment. Methylation percentages were calculated as ratios of the amount of *ZNF671* versus *ACTB* of a sample divided by the same ratio of IVD multiplied by 100.

### Bisulphite pyrosequencing

Bisulphite pyrosequencing of methylation analysis was performed as described previously [57]. In brief, bisulphite-modified DNA (resulting in deamination of unmethylated cytosine to uracil) was subjected to PCR amplification strategy using a tailed reverse primer and a biotin-labeled universal primer (Supplementary Table 1). PCR and sequencing primers were designed using PyroMark Assay Design 2.0 (Qiagen GmbH, Hilden, Germany). A 228-bp fragment (-72 to 156) of the *ZNF671* promoter was PCR-amplified, and pyrosequencing was performed using a PyroMark Q24 (Qiagen) and Pyro Gold Reagents (Qiagen), according to the manufacturer's protocol. The methylation levels of 8 CpG sites, from 41 to 125 relative to the *ZNF671* transcriptional start site (TSS) were measured. The methylation percentage of each cytosine was determined by their fluorescence intensities of cytosines divided by the sum of fluorescence intensity of cytosines and thymines (converted from uracil by PCR) at each CpG site. IVD (*in vitro* methylated DNA) was included as positive control of bisulphite pyrosequencing.

### *In vitro* promoter DNA methylation assay

For the construction of a *ZNF671* promoter plasmid, a 960-bp region of the *ZNF671* promoter (-329/+631) was amplified by PCR using specific primers (Supplementary Table 1) from genomic DNA of HUC cells. The PCR products were then ligated into multiple cloning sites of the pGL3 vector, which was predigested with BglII and NheI. pGL3 vector for sequencing confirmation. For *in vitro* DNA methylation, the *ZNF671* promoter was re-isolated from the pGL3-*ZNF671* promoter by DNA digestion with BglII (New England Biolabs, Beverly, MA) and NheI (New England Biolabs), and *in vitro* methylated using a recombinant CpG methyltransferase (M.SssI, New England Biolabs). The excised fragment without M.SssI methylated treatment was acted as a control. DNA methylation was verified using the methylation-sensitive restriction enzyme, BstUI. Both the methylated and unmethylated promoters were re-liagted into the pGL3 promoter, and 50 ng of ligated DNA was co-transfected with 25 ng *Renilla* luciferase vectors (pRL-TK) into 293T cells in a 24-well plates. Cells were lysed after 48hr and luciferase activities were measured using a Dual Luciferase Reporter Assay System (Promega, Madison, WI) on a luminometer (Turner Designs, Sunnyvale, CA)

### Plasmid constructs and transfection

The complete coding sequence of *ZNF671* was amplified by PCR using specific primers (Supplementary Table 1) from cDNA of HUC cells which endogenously express *ZNF671.* The PCR products were ligated into the multiple cloning site of a pcDNA3.1 mammalian expression vector predigested with KpnI (New England Biolabs) and *BamHI* (New England Biolabs). *ZNF671* expression or empty vectors were transfected into bladder UC UMUC3 cells using Lipofectamine 2000 transfection reagent (Invitrogen) according to the manufacturer's protocol. Transfected cells were cultivated with fresh culture medium containing 800 μg/ml Geneticin (G418, Sigma) and replaced every 3 days. Single colonies that formed were selected for further culture.

### Soft agar colony formation assay

1×10^4^ cells were mixed and seeded in 2ml of 0.3% top agar supplemented with medium and 10% FBS. This suspension was overlaid on the 2.5ml bottom agar of agar of 0.5% agar with medium and 10% FBS in a 60-mm plate. The plates were allowed to solidify and were incubated at 37°C for 14-21 days. At the end of the experiments, colonies were stained with 1mg/ml Iodonitrotetrazolium chloride (INT stain, Sigma) for overnight at 37°C. Colony numbers were counted using Image-Pro 3D Suite software version 5.1.1.38 for Windows (Media Cybernetics, Rockville, MD).

### *In vitro* invasion assays

To assess cell invasion, polycarbonate cell culture inserts (8.0 μm pore size, Merck Millipore, Billerica, MA) were first coated with 25 ul matrigel (25%, BD Biosciences, San Jose, CA) and incubated at 37°C for 30 minutes. 2×10^4^ cells were seeded into the upper chamber of polycarbonate cell culture inserts containing medium with 1% FBS, and then placed into 24 well plate containing medium with 10% FBS. After 48 hours incubation, the cells at the top of the filter were removed by washing with 1X PBS. Cells attached to the bottoms of the membranes were fixed and stained with Giemsa stain (Sigma). Colonies numbers were then counted using Image-Pro 3D Suite software version 5.1.1.38 for Windows (Media Cybernetics).

### *In vivo* tumorigenicity assay

Three athymic nude mice (BALB/cByJNarl, 4-week-old) were obtained from the National Laboratory Animal Center, Taiwan. All mice were kept under specific pathogen-free conditions using a laminar airflow rack with free access to sterilized food and autoclaved water. All experiments were performed under the approval from the Animal Experimentation Ethics Committee of the National Chung Cheng University. 2 × 10^7^ cells of UMUC3 cells stably transfected with pcDNA3.1/*ZNF671* or empty vector were resuspended in 0.1ml of medium and Matrigel (BD Biosciences) mixture (1:1). The cell suspensions were then injected subcutaneously into the left and right flanks of each mouse (day 0). Tumor size was measured daily with calipers in length (L) and width (W). Tumor volumes were calculated using the formula (L x W^2^/2). At the end of the experiment, all mice were sacrificed by cervical dislocation.

### Statistical analysis

Comparisons of non-parametric variables were assessed by Mann-Whitney tests, whenever appropriate. Locoregional disease-free survival was assessed by Kaplan-Meier analysis; curve difference between groups was estimated by the log-rank test. Locoregional disease-free time period was defined as the duration from the day of surgical resection to the day of the detection of new, recurred locoregional lesions by cystoscopy or cross section images (CT or MRI). Multivariate analysis was conducted by Cox hazard-proportional regression analysis. The 95% confidence intervals were provided in conjunction with a point to estimate an effective size. A *ZNF671* methylation at 11%, which is the average *ZNF671* methylation level of low grade tumor samples, was used as a cutoff. All statistical analyses, including ROC curves, were performed using SPSS software version 18.0 (SPSS, Inc., Chicago, IL) or GraphPad Prism 6 (GraphPad Software, Inc., San Diego, CA). *P* < 0.05 was considered statistically significant.

## SUPPLEMENTARY MATERIAL FIGURES AND TABLES



## References

[1] Ploeg M, Aben KK, Kiemeney LA (2009). The present and future burden of urinary bladder cancer in the world. World J Urol.

[2] Siegel R, Ma J, Zou Z, Jemal A (2014). Cancer statistics, 2014. CA Cancer J Clin.

[3] Chen CJ, Chuang YC, Lin TM, Wu HY (1985). Malignant neoplasms among residents of a blackfoot disease-endemic area in Taiwan: high-arsenic artesian well water and cancers. Cancer Res.

[4] Green DA, Rink M, Xylinas E, Matin SF, Stenzl A, Roupret M, Karakiewicz PI, Scherr DS, Shariat SF (2013). Urothelial carcinoma of the bladder and the upper tract: disparate twins. J Urol.

[5] Tan LB, Chang LL, Cheng KI, Huang CH, Kwan AL (2009). Transitional cell carcinomas of the renal pelvis and the ureter: comparative demographic characteristics, pathological grade and stage and 5-year survival in a Taiwanese population. BJU Int.

[6] Williams SG, Stein JP (2004). Molecular pathways in bladder cancer. Urol Res.

[7] Sylvester RJ, van der Meijden AP, Oosterlinck W, Witjes JA, Bouffioux C, Denis L, Newling DW, Kurth K (2006). Predicting recurrence and progression in individual patients with stage Ta T1 bladder cancer using EORTC risk tables: a combined analysis of 2596 patients from seven EORTC trials. Eur Urol.

[8] Prasad SM, Decastro GJ, Steinberg GD (2011). Urothelial carcinoma of the bladder: definition, treatment and future efforts. Nat Rev Urol.

[9] Hansen MF, Cavenee WK (1988). Tumor suppressors: recessive mutations that lead to cancer. Cell.

[10] Hunter T (1997). Oncoprotein networks. Cell.

[11] Jones PA, Baylin SB (2007). The epigenomics of cancer. Cell.

[12] Baylin SB, Jones PA (2011). A decade of exploring the cancer epigenome - biological and translational implications. Nat Rev Cancer.

[13] Seisenberger S, Peat JR, Reik W (2013). Conceptual links between DNA methylation reprogramming in the early embryo and primordial germ cells. Curr Opin Cell Biol.

[14] Laird PW (2003). The power and the promise of DNA methylation markers. Nat Rev Cancer.

[15] Tung PY, Knoepfler PS (2015). Epigenetic mechanisms of tumorigenicity manifesting in stem cells. Oncogene.

[16] Esteller M (2003). Relevance of DNA methylation in the management of cancer. Lancet Oncol.

[17] Chan MW, Chan LW, Tang NL, Tong JH, Lo KW, Lee TL, Cheung HY, Wong WS, Chan PS, Lai FM, To KF (2002). Hypermethylation of multiple genes in tumor tissues and voided urine in urinary bladder cancer patients. Clin Cancer Res.

[18] Chen PC, Tsai MH, Yip SK, Jou YC, Ng CF, Chen Y, Wang X, Huang W, Tung CL, Chen GC, Huang MM, Tong JH, Song EJ, Chang DC, Hsu CD, To KF (2011). Distinct DNA methylation epigenotypes in bladder cancer from different Chinese sub-populations and its implication in cancer detection using voided urine. BMC Med Genomics.

[19] Gonzalez-Zulueta M, Bender CM, Yang AS, Nguyen T, Beart RW, Van Tornout JM, Jones PA (1995). Methylation of the 5′ CpG island of the p16/CDKN2 tumor suppressor gene in normal and transformed human tissues correlates with gene silencing. Cancer Res.

[20] Kandimalla R, van Tilborg AA, Kompier LC, Stumpel DJ, Stam RW, Bangma CH, Zwarthoff EC (2012). Genome-wide analysis of CpG island methylation in bladder cancer identified TBX2, TBX3, GATA2, and ZIC4 as pTa-specific prognostic markers. Eur Urol.

[21] Ibragimova I, Dulaimi E, Slifker MJ, Chen DY, Uzzo RG, Cairns P (2014). A global profile of gene promoter methylation in treatment-naive urothelial cancer. Epigenetics.

[22] Huang da W, Sherman BT, Lempicki RA (2009). Bioinformatics enrichment tools: paths toward the comprehensive functional analysis of large gene lists. Nucleic Acids Res.

[23] Arai E, Chiku S, Mori T, Gotoh M, Nakagawa T, Fujimoto H, Kanai Y (2012). Single-CpG-resolution methylome analysis identifies clinicopathologically aggressive CpG island methylator phenotype clear cell renal cell carcinomas. Carcinogenesis.

[24] Hansel A, Steinbach D, Greinke C, Schmitz M, Eiselt J, Scheungraber C, Gajda M, Hoyer H, Runnebaum IB, Durst M (2014). A promising DNA methylation signature for the triage of high-risk human papillomavirus DNA-positive women. PLoS One.

[25] Cheng Y, Geng H, Cheng SH, Liang P, Bai Y, Li J, Srivastava G, Ng MH, Fukagawa T, Wu X, Chan AT, Tao Q (2010). KRAB zinc finger protein ZNF382 is a proapoptotic tumor suppressor that represses multiple oncogenes and is commonly silenced in multiple carcinomas. Cancer Res.

[26] Cheng Y, Liang P, Geng H, Wang Z, Li L, Cheng SH, Ying J, Su X, Ng KM, Ng MH, Mok TS, Chan AT, Tao Q (2012). A novel 19q13 nucleolar zinc finger protein suppresses tumor cell growth through inhibiting ribosome biogenesis and inducing apoptosis but is frequently silenced in multiple carcinomas. Mol Cancer Res.

[27] Severson PL, Tokar EJ, Vrba L, Waalkes MP, Futscher BW (2013). Coordinate H3K9 and DNA methylation silencing of ZNFs in toxicant-induced malignant transformation. Epigenetics.

[28] Eads CA, Laird PW (2002). Combined bisulfite restriction analysis (COBRA). Methods Mol Biol.

[29] Baylin S, Bestor TH (2002). Altered methylation patterns in cancer cell genomes: cause or consequence?. Cancer Cell.

[30] Kong D, Li Y, Wang Z, Sarkar FH (2011). Cancer Stem Cells and Epithelial-to-Mesenchymal Transition (EMT)-Phenotypic Cells: Are They Cousins or Twins?. Cancers (Basel).

[31] Bedi U, Mishra VK, Wasilewski D, Scheel C, Johnsen SA (2014). Epigenetic plasticity: a central regulator of epithelial-to-mesenchymal transition in cancer. Oncotarget.

[32] McDonald OG, Wu H, Timp W, Doi A, Feinberg AP (2011). Genome-scale epigenetic reprogramming during epithelial-to-mesenchymal transition. Nat Struct Mol Biol.

[33] Pirozzi G, Tirino V, Camerlingo R, Franco R, La Rocca A, Liguori E, Martucci N, Paino F, Normanno N, Rocco G (2011). Epithelial to mesenchymal transition by TGFbeta-1 induction increases stemness characteristics in primary non small cell lung cancer cell line. PLoS One.

[34] Su SF, de Castro Abreu AL, Chihara Y, Tsai Y, Andreu-Vieyra C, Daneshmand S, Skinner EC, Jones PA, Siegmund KD, Liang G (2014). A panel of three markers hyper- and hypomethylated in urine sediments accurately predicts bladder cancer recurrence. Clin Cancer Res.

[35] Witzgall R, O'Leary E, Leaf A, Onaldi D, Bonventre JV (1994). The Kruppel-associated box-A (KRAB-A) domain of zinc finger proteins mediates transcriptional repression. Proc Natl Acad Sci U S A.

[36] Margolin JF, Friedman JR, Meyer WK, Vissing H, Thiesen HJ, Rauscher FJ (1994). Kruppel-associated boxes are potent transcriptional repression domains. Proc Natl Acad Sci U S A.

[37] Urrutia R (2003). KRAB-containing zinc-finger repressor proteins. Genome Biol.

[38] Underhill C, Qutob MS, Yee SP, Torchia J (2000). A novel nuclear receptor corepressor complex, N-CoR, contains components of the mammalian SWI/SNF complex and the corepressor KAP-1. J Biol Chem.

[39] Schultz DC, Friedman JR, Rauscher FJ (2001). Targeting histone deacetylase complexes via KRAB-zinc finger proteins: the PHD and bromodomains of KAP-1 form a cooperative unit that recruits a novel isoform of the Mi-2alpha subunit of NuRD. Genes Dev.

[40] Schultz DC, Ayyanathan K, Negorev D, Maul GG, Rauscher FJ (2002). SETDB1: a novel KAP-1-associated histone H3, lysine 9-specific methyltransferase that contributes to HP1-mediated silencing of euchromatic genes by KRAB zinc-finger proteins. Genes Dev.

[41] Sripathy SP, Stevens J, Schultz DC (2006). The KAP1 corepressor functions to coordinate the assembly of de novo HP1-demarcated microenvironments of heterochromatin required for KRAB zinc finger protein-mediated transcriptional repression. Mol Cell Biol.

[42] Friedman JR, Fredericks WJ, Jensen DE, Speicher DW, Huang XP, Neilson EG, Rauscher FJ (1996). KAP-1, a novel corepressor for the highly conserved KRAB repression domain. Genes Dev.

[43] Moosmann P, Georgiev O, Le Douarin B, Bourquin JP, Schaffner W (1996). Transcriptional repression by RING finger protein TIF1 beta that interacts with the KRAB repressor domain of KOX1. Nucleic Acids Res.

[44] Zheng L, Pan H, Li S, Flesken-Nikitin A, Chen PL, Boyer TG, Lee WH (2000). Sequence-specific transcriptional corepressor function for BRCA1 through a novel zinc finger protein, ZBRK1. Mol Cell.

[45] Huang C, Jia Y, Yang S, Chen B, Sun H, Shen F, Wang Y (2007). Characterization of ZNF23, a KRAB-containing protein that is downregulated in human cancers and inhibits cell cycle progression. Exp Cell Res.

[46] Hu R, Peng G, Dai H, Breuer EK, Stemke-Hale K, Li K, Gonzalez-Angulo AM, Mills GB, Lin SY (2011). ZNF668 functions as a tumor suppressor by regulating p53 stability and function in breast cancer. Cancer Res.

[47] Monteiro-Reis S, Leca L, Almeida M, Antunes L, Monteiro P, Dias PC, Morais A, Oliveira J, Henrique R, Jeronimo C (2014). Accurate detection of upper tract urothelial carcinoma in tissue and urine by means of quantitative GDF15, TMEFF2 and VIM promoter methylation. Eur J Cancer.

[48] Roupret M, Babjuk M, Comperat E, Zigeuner R, Sylvester R, Burger M, Cowan N, Bohle A, Van Rhijn BW, Kaasinen E, Palou J, Shariat SF (2013). European guidelines on upper tract urothelial carcinomas: 2013 update. Eur Urol.

[49] Frietze S, O'Geen H, Blahnik KR, Jin VX, Farnham PJ (2010). ZNF274 recruits the histone methyltransferase SETDB1 to the 3′ ends of ZNF genes. PLoS One.

[50] O'Geen H, Squazzo SL, Iyengar S, Blahnik K, Rinn JL, Chang HY, Green R, Farnham PJ (2007). Genome-wide analysis of KAP1 binding suggests autoregulation of KRAB-ZNFs. PLoS Genet.

[51] Lin LF, Li CF, Wang WJ, Yang WM, Wang DD, Chang WC, Lee WH, Wang JM (2013). Loss of ZBRK1 contributes to the increase of KAP1 and promotes KAP1-mediated metastasis and invasion in cervical cancer. PLoS One.

[52] Cairns P (2007). Gene methylation and early detection of genitourinary cancer: the road ahead. Nat Rev Cancer.

[53] Jeronimo C, Henrique R (2014). Epigenetic biomarkers in urological tumors: A systematic review. Cancer Lett.

[54] Planz B, Jochims E, Deix T, Caspers HP, Jakse G, Boecking A (2005). The role of urinary cytology for detection of bladder cancer. Eur J Surg Oncol.

[55] Christian BJ, Loretz LJ, Oberley TD, Reznikoff CA (1987). Characterization of human uroepithelial cells immortalized *in vitro* by simian virus 40. Cancer Res.

[56] Chou JL, Huang RL, Shay J, Chen LY, Lin SJ, Yan PS, Chao WT, Lai YH, Lai YL, Chao TK, Lee CI, Tai CK, Wu SF, Nephew KP, Huang TH, Lai HC (2015). Hypermethylation of the TGF-beta target, ABCA1 is associated with poor prognosis in ovarian cancer patients. Clin Epigenetics.

[57] Lin HY, Hung SK, Lee MS, Chiou WY, Huang TT, Tseng CE, Shih LY, Lin RI, Lin JM, Lai YH, Chang CB, Hsu FC, Chen LC, Tsai SJ, Su YC, Li SC (2015). DNA methylome analysis identifies epigenetic silencing of FHIT as a determining factor for radiosensitivity in oral cancer: an outcome-predicting and treatment-implicating study. Oncotarget.

