# Supplementary material for: Methylomics analysis identifies ZNF671 as an epigenetically repressed novel tumor suppressor and a potential non-invasive biomarker for the detection of urothelial carcinoma
Source: Oncotarget. 2015 Jul 22;6(30):29555–72. doi: 10.18632/oncotarget.4986 (PMC4745746; doi:10.18632/oncotarget.4986)
Supplement: Supplementary file 1 [file oncotarget-06-29555-s001.pdf]

Methylomics analysis identifies ZNF671 as an epigenetically repressed novel tumor suppressor and a potential non-invasive biomarker for the detection of urothelial carcinoma

Supplementary Material

A

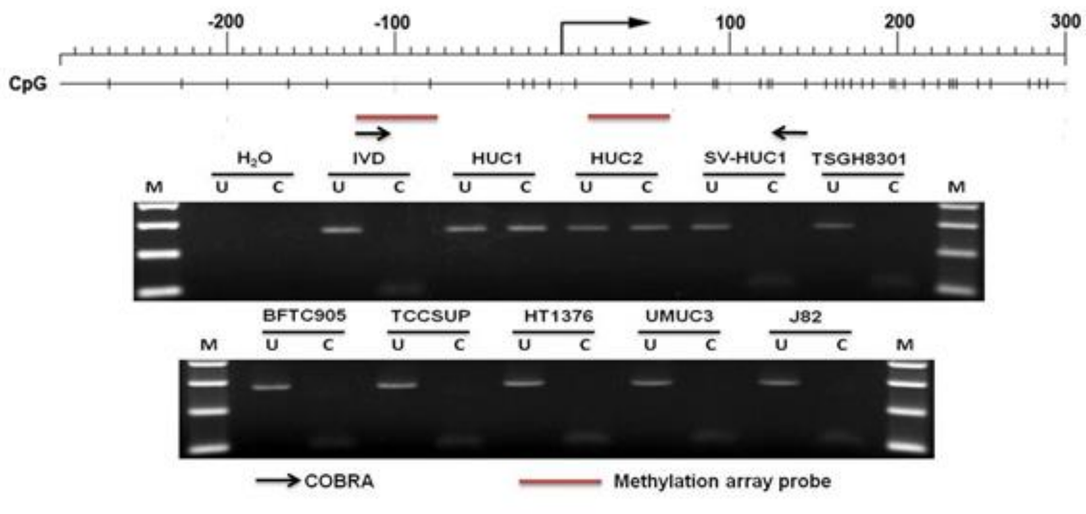

B

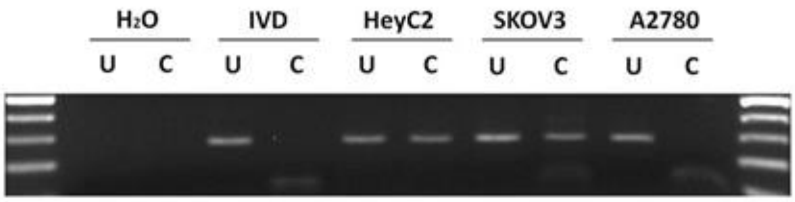

Supplementary Figure 1. DNA methylation analysis of two specific regions of the *ZNF671* promoter (diagrammed in top of A), in various bladder UC (A, lower two panels) vs. ovarian cancer cell lines (B). COBRA ("C") (*co*mbined *b*isulfite *r*estriction *a*nalysis, Xiong and Laird, 1997) was used to assess *ZNF671* methylation status by gel electrophoresis of PCR amplified bisulfite-modified DNA digested with the DNA methylation-sensitive restriction enzyme *AciI*. Undigested control, "U". "M," 100 bp DNA ladder marker. "IVD," (*in vitro* methylation DNA), a COBRA positive control to demonstrate complete digestion.

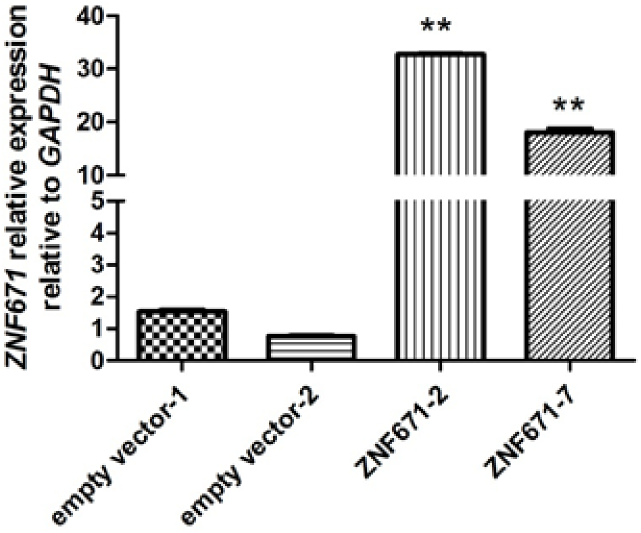

Supplementary Figure 2. *ZNF671* gene expression, relative to that of *GAPDH*, in empty vector- (left two bars) vs. *ZNF*-expressing (right two bars) UMUC3 UC stable cell clones, as determined here by quantitative PCR, were assessed for possible association with soft agar cell survival, in Figure 3. **P<0.01

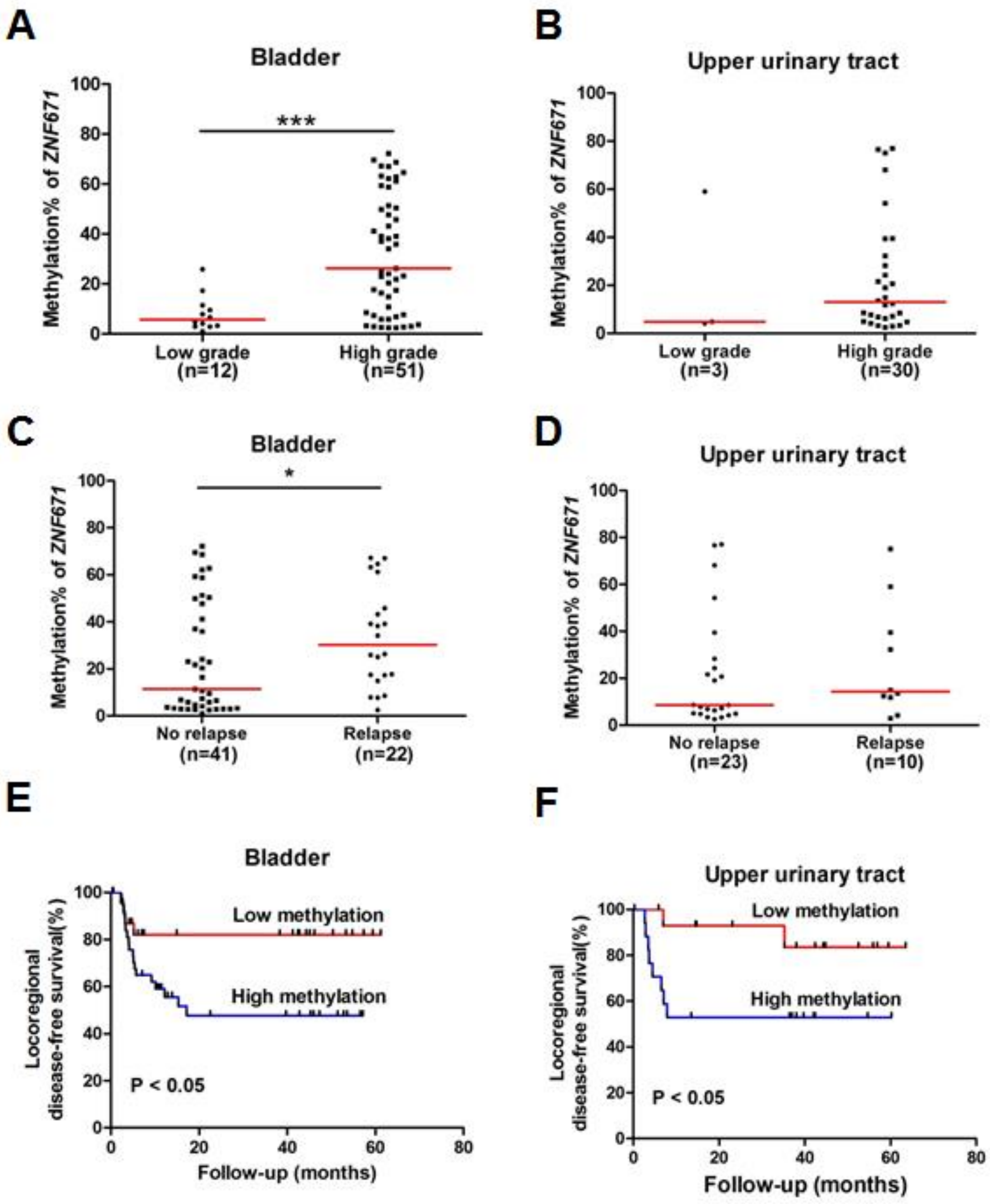

Supplementary Figure 3. *ZNF671* methylation in UC patient tumor tissue samples from bladder or upper urinary tract was determined by bisulphite pyrosequencing. Assessments of association between *ZNF671* methylation and tumor grade (A, B), relapse (C, D), and locoregional disease-free survival (E, F, by Kaplan-Meier analysis) are shown. *P<0.05, ***P<0.01

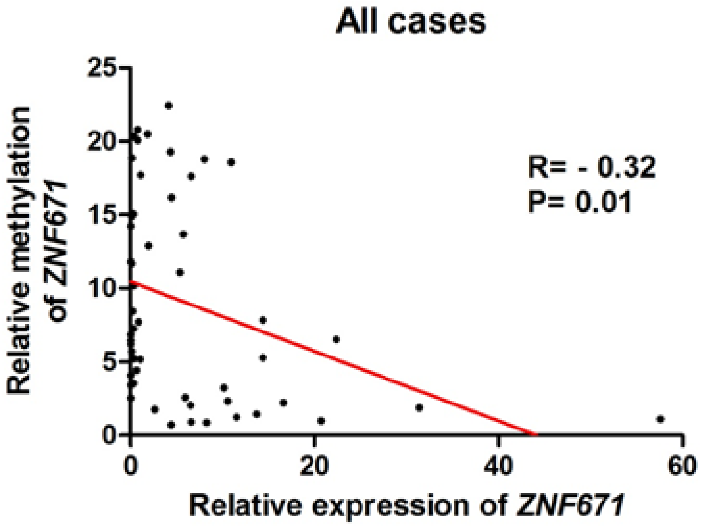

Supplementary Figure 4. Inverse relationship between *ZNF671* expression and methylation levels observed after excluding seven samples (i.e., low expression and low methylation). A positive correlation was observed (Spearman R = -0.32, P=0.01).

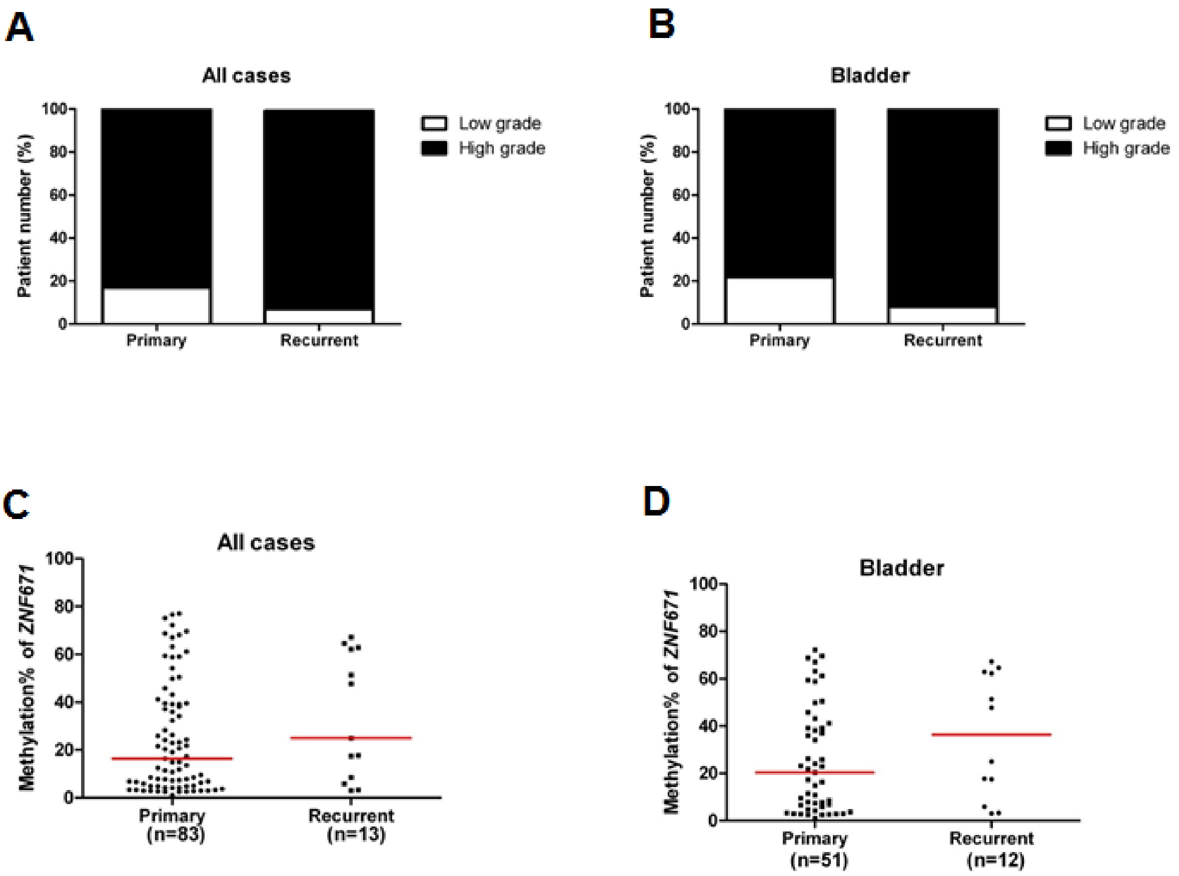

Supplementary Figure 5. Analysis of possible correlation between tumor grade and *ZNF671* methylation in primary or recurrent UC patient tumor tissue samples. Patients with recurrent tumors tended to have higher tumor grade (A, B) and higher *ZNF671* methylation (C, D), as compared to primary tumors. Tumor tissue samples (all cases, n=96, A, C,) or bladder UC patient tumor tissues only (n=63, B, D).

Supplementary Table 1. Primers used in this study

Primer name	Primer sequence (5' to 3')	Annealing Temp (°C)	Product size (bp)
**qMSP**			
ZNF671 MF1	TTTGGTCGGGGTTTTGTAAC	60	100
ZNF671 MR1	AACATCAAACGCGTCTCG		
β-actin (*ACTB*) MF	TGGTGATGGAGGAGGTTTAGTAAGT	60	133
β-actin (*ACTB*) MR	AACCAATAAAACCTACTCCTCCCTTAA		
**COBRA**			
ZNF671 BS-F	TTTTGAAGGATAGTTTAGTGGGAAT	58	272
ZNF671 BS-R	CCATCCCTACAAAACATCAA		
**Bisulphite Pyrosequeincing**			
ZNF671_Pyro-F	ATTTAATGAGGGTTTAGGAGAGG	60	228
ZNF671_Pyro-R	CAAACACTTCCCTCCCTACAAAACAT		
ZNF671_SEQ	GGATATTTTGTTTTTGTTAGGT		
**RT-PCR**			
ZNF671 RT F	GTCATGAAACTAGAGCGAGGAGAA	60	108
ZNF671 RT R	ATGCCAACAACCAGGTCTAAGTC		
GAPDH RT-F	CCCCTTCATTGACCTCAACTACAT	60	135
GAPDH RT-R	CGCTCCTGGAAGATGGTGA		
E-cadherin RT-F	AAACAGGATGGCTGAAGGTG	60	159
E-cadherin RT-R	TGAATTCGGGCTTGTTGTC		
**Plasmid construction**			
ZNF671 cDNA KpnI_F	CGGGGTACCGAGATGTTGTCCCCAGTGTCCC	58	1626
ZNF671 cDNA BamHI_R	CGCGGATCCTTAAAGCTTTTCTCCAGCATGAACCC		
ZNF671 promoter NheI_ F	CTAGCTAGCAGGCGGAGGTTGCAGTCA	62	960
ZNF671 promoter BglII_ R	GGAAGATCTCAGTCCCCACCCCAAACTAA		

Supplementary Table 2. The information of 31 probes in Illumina 27K CpG island methylation array

Target ID	SYMBOL	CHR	Probe locations	GENE_ID	ACCESSION	GID	CPG_ISLAND_LOCATIONS
cg03238797	ADAMTS18	16	16:76026346-76026395	GeneID:170692	NM_139054.2	GI:40806184	16:76025670-76027234
cg15083233	AKAP2	9	9:111850223-111850272	GeneID:11217	NM_001004065.2	GI:51873057	9:111850025-111852175
cg22187630	CACNA1A	19	19:13477871-13477920	GeneID:773	NM_023035.1	GI:13386497	19:13477638-13478603
cg23300372	CART	5	5: 71051188 -71051237	GeneID:9607	NM_004291.2	GI:46852394	5:71050507-71051626
cg08519905	CD9	12	12:6178987-6179036	GeneID:928	NM_001769.2	GI:21237762	12:6178825-6180741
cg10238818	CYYR1	21	21:26867425-26867474	GeneID:116159	NM_052954.2	GI:45827794	21:26866776-26867613
cg01839464	DCC	18	18:48122427-48122476	GeneID:1630	NT_010966.13	GI:4885174	18:48122009-48122893
cg08575537	EPO	7	7:100156514-100156563	GeneID:2056	NM_000799.2	GI:62240996	7:100155958-100156762
cg02489552	FLJ40365	19	19:14982531-14982580	GeneID:126402	NM_173482.1	GI:27735062	19:14982467-14983303
cg21073927	GATA4	8	8:11599516 -11599565	GeneID:2626	NM_002052.2	GI:33188460	8:11598711-11600557
cg26252167	GPR6	6	6: 110407218 -110407267	GeneID:2830	NM_005284.2	GI:31377786	6:110406816-110407975
cg02332525	GRM7	3	3:6878105 -6878154	GeneID:2917	NM_181874.1	GI:32528267	3:6877272-6878818
cg02774439	HAND2	4	4:174687778-174687827	GeneID:9464	NM_021973.1	GI:12545383	4:174686012-174689831
cg11965370	HNT	11	11:131286108-131286157	GeneID:50863	NM_016522.2	GI:38045920	11:131285371-131286756
cg15760840	HOXA11	7	7:27191699-27191748	GeneID:3207	NM_005523.4	GI:24497552	7:27189699-27192279
cg17950095	HOXA11	7	7:27191225-27191274	GeneID:3207	NM_005523.4	GI:24497552	7:27189699-27192279
cg24826867	IRF8	16	16:84490306-84490355	GeneID:3394	NM_002163.2	GI:55953136	16:84489299-84490591
cg16902509	ITGA8	10	10:15801318-15801367	GeneID:8516	NM_003638.1	GI:49170033	10:15801302-15802503
cg17834752	KCNK9	8	8:140784373-140784422	GeneID:51305	NM_016601.2	GI:16445406	8:140783691-140786611
cg03469054	KIAA1944	12	12:128953814-128953863	GeneID:121256	NM_133448.1	GI:42734372	12:128953408-128955096
cg04897683	NEUROG1	5	5:134899361-134899410	GeneID:4762	NM_006161.2	GI:38455395	5:134898284-134900130
cg08441806	NKX6-2	10	10:134449091-134449140	GeneID:84504	NM_177400.1	GI:28912911	10:134447362-134452718
cg09260089	NKX6-2	10	10:134449850-134449899	GeneID:84504	NM_177400.1	GI:28912911	10:134447362-134452718
cg02260587	PCDHB2	5	5:140454432-140454481	GeneID:56133	NM_018936.2	GI:14195608	5:140454244-140454946
cg21176048	PEX5L	3	3:181237881-181237930	GeneID:51555	NM_016559.1	GI:7706670	3:181236808-181238032
cg04490714	SLC6A2	16	16:54248065-54248114	GeneID:6530	NM_001043.2	GI:65506986	16:54246902-54248577
cg16428251	SOX14	3	3: 138966121-38966170	GeneID:8403	NM_004189.2	GI:31563384	3:138964433-138967163
cg04391111	TP73	1	1: 3557864-3557913	GeneID:7161	NT_004321.17	GI:4885644	1:3556965-3559547
cg09053680	UTF1	10	10:134894104-134894153	GeneID:8433	NM_003577.2	GI:71043875	10:134892848-134895213
cg19246110	ZNF671	19	19:62930740-62930789	GeneID:79891	NM_024833.1	GI:13376239	19:62930217-62931307
cg23857226	ZNF671	19	19:62930886-62930935	GeneID:79891	NM_024833.1	GI:13376239	19:62930217-62931307

Supplementary Table 3. Gene Ontology (GO) analysis on molecular function (MF) of the 31 methylated probes using DAVID Bioinformatics Resources 6.7

Term	Gene count	P-values
GO:0030528~transcription regulator activity	10	3.05E-04
GO:0003700~transcription factor activity	8	6.01E-04
GO:0043565~sequence-specific DNA binding	6	2.93E-03
GO:0033613~transcription activator binding	2	1.29E-02
GO:0003677~DNA binding	9	2.37E-02
GO:0016563~transcription activator activity	4	2.73E-02
GO:0022832~voltage-gated channel activity	3	3.91E-02
GO:0005245~voltage-gated calcium channel activity	2	4.44E-02
GO:0003713~transcription coactivator activity	3	4.62E-02
GO:0008134~transcription factor binding	4	4.81E-02

Supplementary Table 4 . Correlation between methylation of *ZNF671* and clinical-pathological data in 96 UC tumor tissue samples

	Bladder (n=63)		Upper urinary tract (n=33)	
	**Methylation (%)**	**P**	**Methylation (%)**	**P**
**Age**				
< 60 years	22.80 ± 23.38[2] (11/63)	0.46	26.00 ± 31.59 (8/33)	0.75
≥ 60 years	28.53 ± 23.33 (52/63)		22.43 ± 21.93 (25/33)	
**Gender**				
Male	27.77 ± 23.25 (51/63)	0.66	23.87 ± 26.65 (17/33)	0.65
Female	26.50 ± 24.26 (12/63)		22.69 ± 21.95 (16/33)	
**Histological Grade[1]**				
Low grade	8.14 ± 7.20 (12/63)	**0.001**	22.70 ± 31.49 (3/33)	0.59
High grade	32.09 ± 23.40 (51/63)		23.36 ± 23.94 (30/33)	
**Pathological Stage**				
pT1	28.68 ± 24.35 (44/63)	0.89	33.84 ± 32.13 (10/33)	0.28
pT2-4	24.85 ± 20.86 (19/63)		18.71 ± 18.73 (23/33)	
**Primary/Recurrent**				
Primary	25.61 ± 22.41 (51/63)	0.19	23.75 ± 24.35 (32/33)	NA
Recurrent	35.69 ± 26.00 (12/63)		8.65125 (1/33)	
**Relapse**				
Yes	33.36 ± 21.18 (22/63)	0.02	26.60 ± 24.47 (10/33)	0.46
No	24.40 ± 23.96 (41/63)		21.86 ± 22.74 (23/33)	
**Methylation**				
Low	4.97 ± 2.63 (24/63)	**<0.001**	5.36 ± 2.04 (15/33)	**<0.001**
High	41.41 ± 18.95 (39/63)		38.24 ± 23.89 (18/33)	

[1]Grading, low grade: G1; high grade: G2-3;

[2]Mean ± SD

Supplementary Table 5. Hazard ratio for locoregional recurrence according to predictive factors in 63 bladder UC tissue samples

	HR(95% CI); P value	
	**Univariate analysis**	**Multivariate analysis**
**Age**		
<60 vs≥60	2.764(0.645-11.836);0.171	NA
**Gender**		
Male vs Female	0.034(0.000-2.898);0.136	NA
**Histological Grade**		
Low vs High grade	1.938(0.572-6.564);0.288	1.488(0.395-5.602);0.557
**Pathological Stage**		
pT1 vs pT2-4	0.587(0.199-1.736);0.336	0.486(0.161-1.467);0.201
**Primary/Recurrent**		
Primary vs Recurrent	1.860(0.684-5.053);0.224	1.773(0.647-4.860);0.266
**Treatment**		
TURBT vs cystectomy	0.045(0.000-271.35);0.486	NA
***ZNF671* Methylation**		
Low vs High	3.029(1.023-8.966);**0.045***	2.753 (0.868-8.727); 0.085

Abbreviation: HR, hazard ratio; 95% CI, 95% confidence interval;
TURBT, trans-urethral resection of bladder tumor

Supplementary Table 6. Hazard ratio for recurrence according to predictive factors in 33 upper urinary tract UC tissue samples

	HR(95% CI); P value	
	**Univariate analysis**	**Multivariate analysis**
**Age**		
< 60 vs ≥60	0.819.(0.211-3.176);0.773	NA
**Gender**		
Male vs Female	1.063(0.307-3.679);0.923	NA
**Histological Grade**		
Low vs High grade	0.343(0.072-1.637);0.179	0.078(0.09-0.707);**0.023***
**Pathological Stage**		
pT1 vs pT2-4	1.585(0.336-7.474);0.561	3.716(0.524-26.33);0.189
**Primary/Recurrent**		
Primary vs Recurrent	0.047(0.000-76764.23);0.675	NA
**ZNF671 Methylation**		
Low vs High	4.473(0.948-21.104);0.058	7.889(1.323-47.058); **0.023***

Abbreviation: HR, hazard ratio; 95% CI, 95% confidence interval;
TURBT, trans-urethral resection of bladder tumor
